# Hypermobility Among Adolescents and the Association With Spinal Deformities: A Large Cross-Sectional Study

**DOI:** 10.5435/JAAOSGlobal-D-24-00047

**Published:** 2024-07-10

**Authors:** Oded Hershkovich, Barak Gordon, Estela Derazne, Dorit Tzur, Arnon Afek, Raphael Lotan

**Affiliations:** From the Department of Orthopedic Surgery, Wolfson Medical Center, Holon, Israel (Dr. Hershkovich and Dr. Lotan); the Medical Corps, Israeli Defense Forces, Israel (Dr. Gordon, Derazne, and Tzur); the Faculty of Medicine, Tel Aviv University, Tel Aviv, Israel (Dr. Hershkovich, Dr. Afek, and Dr. Lotan); and the Chaim Sheba Medical Center, Tel Hashomer, Israel (Dr. Afek).

## Abstract

**Introduction:**

Adolescent idiopathic scoliosis and Scheuermann kyphosis are common spinal deformities (SD) among adolescents. The potential link between hypermobility and SD is a topic of debate. We aimed to investigate the prevalence of hypermobility and its association with SD.

**Methods:**

A cross-sectional analysis of records of 17-year-old subjects who were recruited into mandatory military service was conducted. Study population comprised 1,220,073 subjects. Prevalence rates were calculated for hypermobility and different categories of SD by severity, studying the strength of the association between hypermobility and SD.

**Results:**

Of 1,220,073 subjects, 0.0111% exhibited hypermobility. Spinal deformities were identified in 10.5% of subjects. Specifically, 7.9% had mild SD, 2.4% had moderate SD, and 0.1% had severe SD. The overall association between hypermobility and SD showed an odds ratio of 2.31 (*P* < 0.001). Subgroup analyses revealed ORs of 1.226 (*P* = 0.041) for mild deformities, 5.783 (*P* < 0.001) for moderate deformities, and 4.01 (*P* = 0.002) for severe deformities. The association was stronger for moderate and severe SD.

**Conclusions:**

This study establishes a notable association between hypermobility and SD among adolescents. The findings highlight the importance of understanding this relationship, which could contribute to advancements in comprehending SD development. Additional research is warranted to expand upon these findings.

Spinal deformities (SD) in adolescents, primarily adolescent idiopathic scoliosis (AIS) and Scheuermann kyphosis (SK), are widely studied. Adolescent idiopathic scoliosis is the most frequently observed type of scoliosis, manifesting after age 10 and occurring in 1% to 3% of the population.^1^ Spinal deformity manifests in several ways: even minimal curvatures have been linked to back pain experienced during adolescence and subsequent years,^2^ notable psychosocial well-being, body image perception,^3^ and, in severe cases, may impair respiratory functions.^4^

The cause and development of scoliosis and kyphosis remain enigmatic, although multiple factors likely influence these. Certain elements such as increased height, late onset of puberty, especially delayed menarche in females, and a diminished body mass index have been identified as contributors.^5,6,8^ Screening strategies encompass Adam's forward bend test, scoliometer measurements of trunk rotation angle, and back surface topography that harnesses digital imagery to detect spinal anomalies.^9^

Hypermobility, a condition where the joints can move beyond the typical range, is observed in varying degrees across populations.^10,11,12^ While hypermobility pertains to the laxity of ligaments and joint capsules, hyperflexibility describes the excessive stretching capabilities of the musculotendinous unit. The two, although distinct, often manifest with overlapping symptoms and can be seen concurrently in individuals.^13^ In previous studies, hypermobility has been associated with low back pain,^14,15^ possibly due to excessive spinal segment motion.

The prevalence of musculoskeletal hypermobility varies widely, from 7% to 59% in adolescents.^16,17^ Hypermobility is more frequently observed in women and typically diminishes with advancing age^18^ and presents a spectrum from benign hypermobility to symptomatic hypermobility disorders, including joint sounds and musculoskeletal discomfort.^19,20^ The Beighton score, evaluating mobility across nine joints, is a prevalent measurement of hypermobility.^21,22^ Typically, a score of 4/9 or above indicates generalized musculoskeletal hypermobility. However, this threshold might overly emphasize clinically significant hypermobility.^23^

Both spinal deformity and musculoskeletal hypermobility are characteristics found in inherited conditions like Marfan syndrome, osteogenesis imperfecta, and specific variants of Ehlers-Danlos syndrome, all of which have notable mutations in connective tissue genes.^24,25^ This coexistence hints at a potential shared causal mechanism between hypermobility and idiopathic scoliosis, where excessive flexibility and rotation of an evolving spine might play a role in AIS's emergence. However, the debate surrounding the association between hypermobility and SD in adolescents remains inconclusive. There exists a paucity of literature that delves deep into this possible connection. Some argue that hypermobility's excessive range of motion might predispose individuals to abnormal spinal movements, potentially leading to deformities.^26,27^ Meanwhile, others posit that the biological mechanisms linked with hypermobility might be a driving factor in the onset or exacerbation of SD, such as in Ehler-Danlos or Marfan syndromes.^28,29^‏

Given the gaps in the current literature and the potential implications of establishing a link between SD and hypermobility, our study aims to shed light on this issue. We aimed to quantify the prevalence of hypermobility in adolescents and assess its potential association with adolescent SD, including mainly idiopathic scoliosis and SK. Such insights will contribute to the broader understanding of these conditions and may guide future preventive and therapeutic strategies.^8^

## Methods

At 17 years of age, most male and female Israelis are mandated by law to undergo a comprehensive medical evaluation at a military recruitment center for medical classification before recruitment. This is an imperative step before their enlistment into mandatory military service. This evaluative process is thorough and entails the completion of a medical questionnaire by the individual, supplemented with a medical report corroborated by their primary care physician. The designed questionnaire is comprehensive, touching upon various aspects such as musculoskeletal history (including fractures, dislocations, and sprains) and history of rheumatic diseases.

After this, the candidates are subjected to a meticulous anamnesis and a physical examination conducted by trained army medical personnel. Based on the initial assessment, they might be directed to specialized medical consultants or recommended additional imaging diagnostics. At the culmination of these evaluations, each individual is ascribed a specific code on a universal medical profile scale. These codes, in essence, encapsulate their medical diagnoses and overall health status. These categorizations align with the Regulations of Medical Fitness Determination, delineating myriad medical conditions.

The study's data were extracted from the Israeli Medical Corps database, as approved by the Israel Defense Forces Medical Corps Institutional Review Board. Owing to the epidemiological nature of this study, there was no requirement for informed consent.

This is a retrospective epidemiological study with complete medical data, including 1,220,073 adolescents evaluated by regional army recruitment centers since 1998.

For a more granulated analysis, subjects with documented SD were classified based on specific disability codes associated with the Regulations of Medical Fitness Determination. This categorization was further bifurcated based on the presence or absence of hypermobility. All subjects diagnosed with SD were classified into one of three severity groups: “Mild' indicated balanced SD with no clinical complaints, kyphosis or scoliosis with no complaints, and a standing radiograph showing a scoliosis curve less than 20° or a kyphosis curve less than 45°. A ‘Moderate’ deformity indicated balanced SD with clinical complaints, kyphosis or scoliosis with clinical complaints, and a standing radiograph that showed a scoliosis curve greater than 20° and less than 50° or a kyphosis curve less than 70° and greater than 45°. A ‘Severe’ deformity indicated severe balanced or unbalanced SD with clinical complaints and with possible functional disability (eg, neurological deficit or respiratory complaints) with a standing radiograph that showed a scoliosis curve greater than 50° (balanced or not) or a kyphosis curve greater than 70°.^30^

Furthermore, the assessment of hypermobility was robust. It involved a two-pronged approach: The initial screening through the self-reported medical questionnaire, highlighting hypermobility and associated clinical manifestations like joint pain and recurrent sprains. This was substantiated with a subsequent physical evaluation using the Beighton scoring system.^21,31,32^ Only scores of 5/9 or higher were considered indicative of hypermobility. It is essential to note that inherent hypermobility conditions, such as Marfan disease and Ehlers-Danlos syndrome, were consciously excluded from the evaluation based on recruits' medical records.

The statistical analyses revolved around discerning potential associations between SD and hypermobility. Logistic regression was used, with data being modelled as binary and multinomial sets. The logistic regression results were elucidated using odds ratios, their respective 95% confidence intervals, and *P* values. All analytical procedures were executed using SPSS software, version 19.0.

## Results

### Prevalence of Hypermobility and Spinal Deformities

Of the cohort of 1,220,073 adolescents, the prevalence of hypermobility was found to be fairly low, with only 1,355 subjects, or 0.01% of the cohort, identifying with this condition. By stark contrast, SD were more frequent; 128,282 subjects had some form of spinal deformity, summing to 10.5% of the cohort. A breakdown of this segment revealed that the majority, 96,950 subjects (7.9%), presented with mild SD. The prevalence of moderate SD was noted in 29,539 subjects (2.4%). Other 1,793 subjects (0.1%) were diagnosed with severe SD, as ascertained by the RFMD. Table 1 provides a detailed comparative overview of the prevalence of hypermobility vis-à-vis the severity of SD.

**Table 1:** Hypermobility and the Prevalence of Spinal Deformities by Severity in Adolescents

	Total	No Spinal Deformities		All Spinal Deformities		Mild spinal Deformities		Moderate Spinal Deformities		Severe Spinal Deformities	
		No	%	No	%	No	%	No	%	No.	%
No hypermobility	1,218,718	1,090,725	89.5%	127,993	10.5%	96,834	7.9%	29,373	2.4%	1786	0.1%
Hypermobility	1355	1066	48.7%	289	21.3%	116	8.6%	166	12.3%	7	0.5%
Total	1,220,073	1,091,791	89.5%	128,282	10.5%	96,950	7.9%	29,539	2.4%	1793	0.1%

The prevalence of SD in our study was 10.5%, which is within the reported incidence expected when combining the prevalence of AIS and SK, according to previous studies.^30^

### Association Between Hypermobility and Spinal Deformities

The pivotal aspect of this study was to discern any potential association between hypermobility and SD. Our statistical models (Table 2) revealed notable associations. The OR for all SD with hypermobility stood at 2.31 (95% CI: 2.03 to 2.63, *P* < 0.001), suggesting that subjects with hypermobility had over twice the risk of developing any spinal deformity than their nonhypermobility counterparts.

**Table 2:** Odds Ratio and 95% CI for Spinal Deformities in Relation to Hypermobility in Adolescents

	All Spinal Deformities^a^			Mild Spinal Deformities^b^			Moderate Spinal Deformities^b^			Severe Spinal Deformities^b^		
	OR^a^	95% CI	*P*	OR^b^	95% CI	*P*	OR^b^	95% CI	*P*	OR^b^	95% CI	*P*
Odds ratio for hypermobility	2.31	2.03-2.63	<0.001	1.23	1.01-1.49	*P* = 0.04	5.78	4.91-6.83	*P* < 0.001	4.01	1.90 to 8.45	*P* = 0.002

CI = confidence interval, OR = odds ratio

a Odds ratios from binary logistic regression.

b Odds ratios from multinomial logistic regression with no low back pain as the base category.

When diving deeper into the specific severity of SD, the results were as follows: For mild SD, the OR was 1.23 (95% CI: 1.01 to 1.49, *P* = 0.041). For moderate SD, the OR surged to 5.78 (95% CI: 4.91 to 6.83, *P* < 0.001). For severe SD, the OR was calculated at 4.01 (95% CI: 1.90 to 8.45, *P* = 0.002). These findings indicate a progressive increase in the association of hypermobility with the severity of SD, peaking at the moderate stage. Although still robust, the association slightly tapers off for severe deformities but remains markedly high.

## Discussion

This investigation delved deep into the intricate relationship between hypermobility and SD. While SD, including AIS and SK, are among the most common spinal abnormalities observed among adolescents, our study's foundational question revolved around the possible linkage of hypermobility to these deformities. This topic has elicited much debate in the scientific literature.

Our data showcased a marked association between hypermobility and SD. The odds ratios indicated a progressive association that peaked at moderate deformities, suggesting that hypermobility, more than merely an interesting clinical observation, might play a crucial role in developing or exacerbating SD.

The pathogenesis of SD remains contentious, and our findings add another layer of complexity. The robust association between hypermobility and SD, especially the pronounced association with moderate and severe deformities, aligns with the potential biomechanical implications hypermobility may have. As the introduction hinted, hypermobility might increase mechanical loads on the spine, potentially leading to earlier degenerative changes through excessive load-bearing. The markedly higher risk of moderate and severe deformities among patients with hypermobility underscores this hypothesis.

The vast difference in prevalence between hypermobility (0.01%) and SD (10.5%) is noteworthy. Although only a fraction of the study population exhibited hypermobility, their increased risk of developing SD, especially of moderate and severe grades, is crucial for clinical considerations. Such data suggest that although hypermobility in isolation may be rare, its association with moderate to severe deformity can be disproportionately high, probably demanding attention to curve progression and additional investigation of clinical significance. Mild spinal deformity, that is, AIS with Cobb angles less than 25°, with hypermobility may warrant a more aggressive nonsurgical treatment and closer follow-up.

This study has several limitations, including the retrospective design that might be prone to biases related to data recording and selection. The study relied heavily on the Beighton score for hypermobility diagnosis; although widely accepted, other diagnostic tools might provide different insights. Our study, by design, delineates an association between hypermobility and spinal deformity but not causation. Other unmeasured confounding variables might play a role in the observed associations. Another possible limitation may be the exclusion of conditions like Marfan disease and Ehlers-Danlos syndrome. These influence joint flexibility and could provide additional insights if included.

Our findings forge a bridge connecting hypermobility to SD, emphasizing the need to consider hypermobility as a potential risk factor. However, the pathophysiological basis requires elucidation. Considering our findings and limitations, we advocate for prospective studies to delve deeper into this connection and possibly guide targeted interventions in the future.

## Conclusions

The relationship between hypermobility and SD, particularly among adolescents, has been a subject of ongoing debate. Our study elucidates a pronounced association between these two conditions, highlighting the heightened risk of developing moderate and severe SD in individuals with hypermobility.

Given the disproportionate prevalence of hypermobility (0.01%) in the population in contrast to the substantially higher occurrence of SD (10.5%), it is evident that hypermobility, although less frequent, plays a notable role in the progression of spinal complications.

Clinically, this underscores the importance of early identification and, possibly, intervention for adolescents with hypermobility to mitigate potential SD. The evidence also suggests a need for tailored management and preventive strategies for this subpopulation.

Additional prospective investigations are paramount to validate these findings, elucidate the underlying pathophysiology, and develop more refined and evidence-based interventions to prevent or ameliorate hypermobility-related SD.
